# Does the weekend matter? evaluating the weekend effect on pregnancy outcomes in first-cycle fresh IVF/ICSI embryo transfers: a retrospective study

**DOI:** 10.1186/s12884-025-07783-x

**Published:** 2025-07-03

**Authors:** Tianli Yang, Yuanyuan Yang, Zhaojuan Hou, Tianli Chang, Nenghui Liu, Donge Liu, Yumei Li, Jing Zhao, Qiong Zhang, Zhongyuan Yao, Fen Tian, Yanping Li

**Affiliations:** 1 Reproductive Medicine Center, Xiangya Hospital of Central South University, 87 Xiangya Road, Changsha, Hunan 410008 People’s Republic of China; 2 Clinical Research Center for Women’s Reproductive Health in Hunan Province, Changsha, Hunan 410008 People’s Republic of China

**Keywords:** Infertility, Weekend effect, Clinical pregnancy rate, Live birth rate, Oocyte retrieval, Embryo transfer, Assisted reproductive technology

## Abstract

**Background:**

The weekend effect is a controversial epidemiological phenomenon that has rarely been reported in the assisted reproductive technology (ART) setting, particularly among first-cycle fresh embryo transfer (ET) populations undergoing in vitro fertilization (IVF) or intracytoplasmic sperm injection (ICSI).

**Methods:**

This was a retrospective cohort study including first fresh ET cycles in the Reproductive Medicine Center, Xiangya Hospital of Central South University from January 1, 2014 to March 1, 2022. Patients were divided into three groups based on the day of oocyte retrieval: Weekend Group, Near-Weekend Group and Midweek Group. Univariable and multivariate logistic regression analyses were performed to identify confounding factors and to evaluate associations between procedural timing and clinical pregnancy rate (CPR).

**Results:**

A total of 8,200 first fresh ET cycles qualified for analysis after exclusions (16,121 screened), and were categorized based on the day of oocyte retrieval: Weekend Group (Saturday/Sunday, 27.06%, *n* = 2,219), Near-Weekend Group (Friday/Monday, 29.70%, *n* = 2,435), and Midweek Group (Tuesday/Wednesday/Thursday, 43.24%, *n* = 3,546). Oocyte retrievals near the weekend significantly reduced the odds of achieving CPR compared to midweek group (adjusted OR: 0.836, 95%CI: 0.728–0.960, *p* = 0.011). Weekend retrievals also showed lower odds of CPR, though not statistically significant (adjusted OR: 0.900, 95%CI: 0.781–1.037, *p* = 0.144). Compared to midweek oocyte retrievals, both the Weekend and Near-Weekend Groups demonstrated significantly lower live birth rates (46.73% vs. 43.88% vs. 43.49%, respectively; *p* = 0.023).

**Conclusions:**

The timing of oocyte retrieval near weekends was associated with reduced CPR and live birth rate in fresh cycles. This study highlights the importance for maintaining consistent clinical vigilance—regardless of the day of the week—to optimize both pregnancy success rates and patient satisfaction.

**Supplementary Information:**

The online version contains supplementary material available at 10.1186/s12884-025-07783-x.

## Introduction

Despite advances in assisted reproductive technology (ART), inconsistencies in pregnancy success rates remain a concern. This variability may be attributed to the biological heterogeneity of infertile couples [1, 2] and disparities in procedural approaches across clinics [3]. While considerable efforts have been made to optimize pregnancy success [4], the timing of ART procedures has not been scrutinized as thoroughly as other aspects. Notably, the “weekend effect”, which refers to the differences in provision of clinical practice between weekends and weekdays [5], poses a unique challenge and may result in varied prognostic outcomes.

The weekend effect is a controversial epidemiological phenomenon in which some studies have reported worse clinical outcomes for both surgical [6] and non-surgical [7, 8] populations admitted during weekends, while others have failed to observe this effect [9]. This phenomenon has been extensively investigated across various fields of medicine, encompassing diverse patient populations and clinical practice patterns. However, in obstetrics and particularly in ART, only a limited number of studies with relatively small sample sizes have directly examined the weekend effect [10, 11]. In the recent largest study, no significant differences were observed in the core outcomes between weekday and weekend/holiday procedures, despite a higher average workload per embryologist on weekends and holidays [12].

At numerous ART centers worldwide, oocyte retrieval and embryo transfer are routinely scheduled exclusively on weekdays, regardless of the ovarian stimulation protocol used [13]. In contrast, most reproductive clinics in China maintain consistent staffing and provide uninterrupted medical services throughout the week, including weekends. Our reproductive medicine center is one of the clinical departments of Xiangya Hospital, a major tertiary teaching hospital affiliated with Central South University (CSU) in central southern China. Given both the paucity of published data and the operational discrepancies in ART procedures between Western and Chinese clinical settings, this retrospective study aimed to investigate the potential existence of a weekend effect in ART outcomes based on our institutional data.

## Materials and methods

### Study design and setting

The study was a retrospective cohort included first fresh embryo transfer cycles at the Reproductive Medicine Center, Xiangya Hospital of CSU from January 1, 2014 to March 1, 2022.

To avoid introducing additional complexities, we only included infertile women aged 20–40 years with a body mass index (BMI) between 18.5 and 27.9 kg/m^2^ undergoing their first embryo transfer cycles. Additionally, we applied a series of relatively stringent exclusion criteria:

1. endometrial pathologies: intrauterine adhesion (IUA), endometrial polyps, chronic endometritis (CE), hyperplasia, tuberculosis, submucous myoma and others;
2. uterine pathologies: uterine malformation, fibroids or adenomyoma, adenomyosis, stage III-IV endometriosis (EMs);
3. pelvic abnormalities: hydrosalpinx, pelvic tuberculosis;
4. comorbidities: diabetes mellitus (DM), hypertension, malignant tumors, etc.;
5. frozen-thawed embryo transfer cycles;
6. blastocyst-stage embryo transfer cycle.

**Fig. 1:**
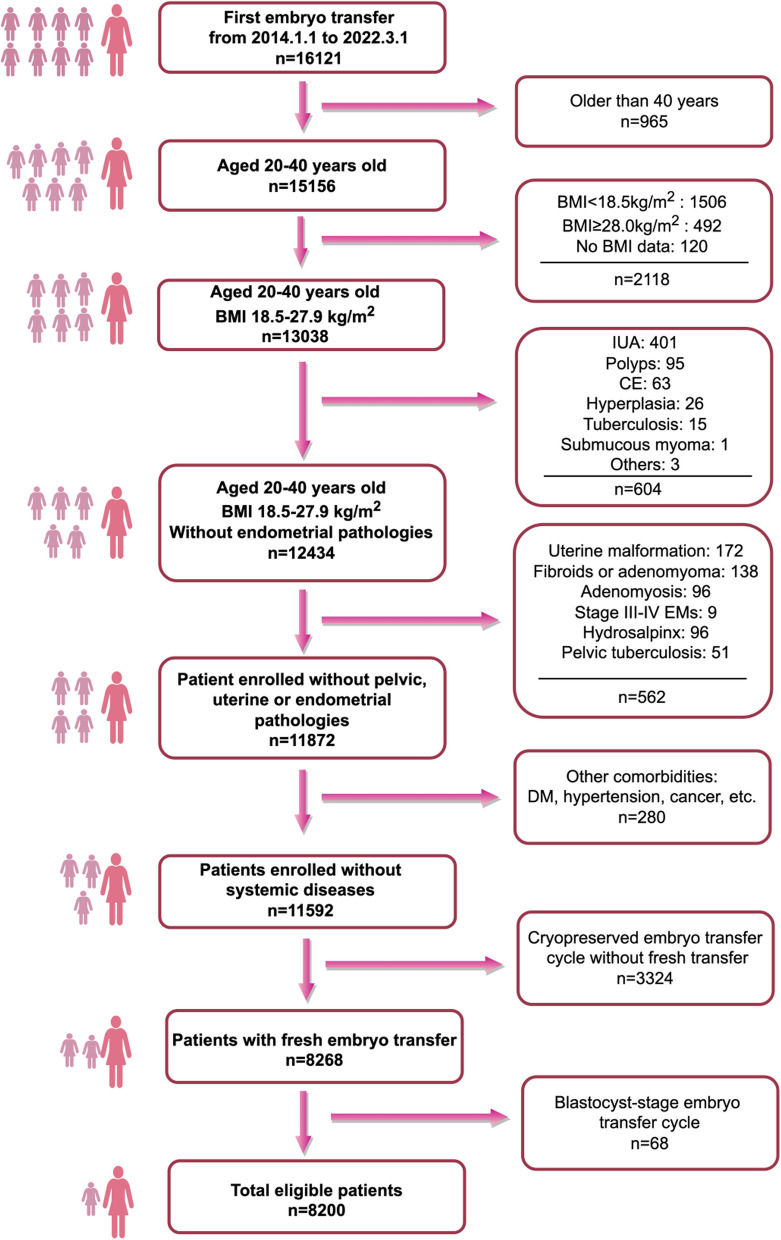
Flowchart for patient screening. IUA, intrauterine adhesion; CE, chronic endometritis; EMs, endometriosis; DM, diabetes mellitus

A total of 16,121 patients who underwent their first embryo transfer between January 1, 2014 and March 1, 2022 were initially screened. After applying the inclusion and exclusion criteria, 8,200 patients were included in the final analysis. The flowchart for patient screening is depicted in Fig. 1.

### Ethics statement

Clinical data of eligible participants were obtained from the Clinical Reproductive Medicine Management System database (CCRM, Nanjing Designing-Future Information System Co., Ltd, China). Details pertaining to routine clinical practice, including timing of procedures or medication dosages, along with follow-up records on pregnancy outcomes, were comprehensively documented within the system.

Given the retrospective nature of this study utilizing anonymized clinical data, the requirement for individual informed consent was formally waived by the Institutional Review Board (IRB) of the Reproductive Medicine Center, Xiangya Hospital, CSU (Approval No. 2024002; Approval Date: July 10, 2024). This exemption aligns with national regulations and the ethical guidelines of the Declaration of Helsinki (2013 revision) for retrospective analyses involving pre-existing, de-identified data. All patient data were rigorously anonymized during collection and analysis, with identifiers removed to protect confidentiality. The study protocol strictly complied with institutional data protection policies, and access to raw datasets was restricted to authorized investigators. Ethical oversight was maintained throughout the research process to ensure adherence to principles of minimal risk and maximal privacy protection.

### Exposures of interest

The present study specifically focused on one of the most critical procedures for assisted reproductive clinicians, i.e., oocyte retrieval. We retrieved the procedure dates from the database. For fresh embryo transfer cycles, once the oocyte retrieval day is determined, the embryo transfer day is generally also fixed. In our center, Day 3 cleavage-stage embryos are typically transferred. As such, all fresh embryo transfer cycles were stratified into three distinct groups based on the day of oocyte retrieval:

- Group 1 (Weekend Group): cycles in which oocyte retrieval performed on Saturday or Sunday (*n* = 2,219);
- Group 2 (Near-Weekend Group): cycles in which oocyte retrieval conducted on Friday or Monday (*n* = 2,435);
- Group 3 (Midweek Group): cycles in which oocyte retrieval occured on Tuesday, Wednesday, or Thursday (*n* = 3,546).

### Ovarian stimulation

The ovarian stimulation protocol was individualized, as thoroughly described in our previous publication [14]. Briefly, two protocols utilized the short-acting gonadotropin-releasing hormone (GnRH) agonist triptorelin (Jinsai, China). For the long protocol, 0.05–0.10 mg of triptorelin was administered daily beginning 7 days after ovulation and continued for 14 days. For the short protocol, triptorelin administration commenced on menstrual cycle Day 2 and continued until the day of hCG administration.

For the long-acting GnRH agonist protocol, a single 3.75-mg dose of leuprorelin acetate (Enantone; Takeda, Japan) was administered on menstrual cycle Day 2, with gonadotropin (Gn) initiation 30 days later. For the ultra-long protocol, 3.75 mg of leuprorelin was readministered after 28 days after the first dose, followed by Gn initiation 21 days thereafter.

Exogenous recombinant human follitropin (Jinsaiheng, Jinsai, China) and/or menotropin (Lebaode, Lizhu, China) were injected to induce follicular development. The initial Gn dosage ranged from 112.5 to 300.0 IU/day, tailored to maternal age and ovarian reserve parameters. Subsequent adjustments were made based on follicular growth dynamics during the stimulation.

In the GnRH antagonist protocol, 0.25 mg of cetrorelix (Cetrotide; Merck Serono, Germany) was administered once the leading follicle reached 12–14 mm in diameter and serum estradiol (E2) levels exceeded 150–400 pg/ml.

For patients with poor ovarian reserve, we adopted the progestin-primed ovarian stimulation (PPOS) protocol. Beginning on menstrual cycle day 3, daily Gn injections were initiated concurrently with oral progesterone capsules (200 mg/day; Qining, Aisheng, China), with both continued until the day of hCG trigger.

### Oocyte triggering, retrieval and insemination

The triggering criteria were defined as follows: presence of at least one dominant follicle ≥ 18 mm, or two dominant follicles ≥ 17 mm, or three dominant follicles ≥ 16 mm, accompanied by an average estradiol (E2) concentration of 200–300 pg/ml per mature follicle. Ovulation was induced by an intramuscular injection of 6000–10000 IU of human chorionic gonadotropin (hCG, Lizhu, China) administered at 21:00. In cases with a heightened risk of ovarian hyperstimulation syndrome (OHSS), the procedure was modified by either reducing the hCG dose or utilizing a dual trigger strategy (i.e., combining hCG with a GnRH agonist).

Oocytes were retrieved 36 h later under the guidance of transvaginal ultrasonography by experienced reproductive endocrinologists, followed by conventional in vitro fertilization (IVF) or intracytoplasmic sperm injection (ICSI) performed in standard fashion. Approximately 6 h after IVF, early rescue ICSI was performed on unfertilized oocytes at the metaphase II (MII) stage if the fertilized oocytes was less than 50%.

### Fresh embryo transfer

In our center, fresh embryo transfers primarily involve cleavage-stage embryos, with blastocyst transfers reserved for select cases. Cleavage-stage embryos were graded according to the ASEBIR embryo assessment criteria [15] and transferred on Day 2 or Day 3 post-oocyte retrieval based on embryonic quality. Blastocysts were evaluated according to Gardner’s criteria [16] and transferred on Day 5 or Day 6 after oocyte retrieval. Standard luteal support was administered using oral progesterone capsules at a dose of 200 mg/day and vaginal micronized progesterone (Utrogestan, Besins, France) at a dose of 600 mg/day, beginning on the day of oocyte retrieval.

### Follow-up

Serum β-hCG, E2, and P4 levels were measured 12 days after embryo transfer to determine pregnancy status. When β-hCG levels exceeded 100 IU/ml, indicating early pregnancy, luteal support was maintained. Clinical pregnancy was confirmed via transvaginal ultrasound at 28 days post-embryo transfer, with follow-up ultrasonography performed one week later. Subsequent pregnancy outcomes (including delivery and neonatal parameters) were monitored through structured telephone follow-up.

### Outcome of interest

The primary outcome was the clinical pregnancy rate (CPR), defined as the presence of intrauterine gestational sac(s) confirmed by ultrasound 4–5 weeks after embryo transfer per total embryo transfer cycles.

Secondary outcomes included:

1. implantation rate (number of gestational sacs divided by the number of embryos transferred);
2. biochemical pregnancy rate (serum β-hCG > 100 IU/L without subsequent ultrasound confirmation);
3. miscarriage rate (pregnancy loss < 28 weeks among intrauterine pregnancies);
4. ongoing pregnancy rate (viable pregnancies beyond 12 weeks);
5. live birth rate (deliveries ≥ 20 gestational weeks per ET cycle).

All ultrasound confirmations were performed by experienced sonographers using standardized protocols.

### Staffing

Our institution maintains year-round, seven-day medical service availability, with closures occuring only during the Spring Festival (the most significant traditional Chinese holiday). No adjustments were made to ovarian stimulation protocols to accommodate weekend days. Clinical practice is supported by four dedicated medical teams, each comprising a senior physician (associate chief physician or higher), an attending physician, and four to five medical assistants.

During weekdays, all physicians are on duty to ensure continuous patient care and comprehensive management. On weekends, a condensed morning schedule (8:00 a.m. to 12:00 p.m.) is implemented, with two rotating physicians covering clinical duties. This scheduling model provides approximately 1.5 rest days per week for physicians, balancing workload and well-being. Each medical assistant receives two full days off per week.

The embryology laboratory is staffed by a team of nine qualified embryologists and three trained medical assistants, with clearly defined responsibilities. While embryologists perform all specialized technical procedures, medical assistants conduct rigorous double-verification of patient identity and sample matching. A rotational shift system ensures continuous laboratory coverage, with seven embryologists and two medical assistants on duty each day, providing every staff member with 1–2 rest days per week to support operational sustainability and staff well-being.

The nursing team comprises nine registered nurses with specialized training in reproductive medicine. In addition to standard clinical nursing responsibilities, their duties encompass comprehensive medical record management and structured telephone follow-ups. During weekdays, all team members provide morning coverage, while afternoon shifts follow a rotational schedule that maintains six to seven staff on duty. Weekend scheduling ensures each nurse receives one to two consecutive off-duty days every two weeks.

### Statistical analysis

Statistical analyses were performed using SPSS (version 25.0; IBM Corp., Armonk, NY, USA). Continuous variables were described as the mean ± standard deviation (SD) for normally distributed data or as the median and interquartile range [interquartile range (IQR)] for non-normally distributed data, assessed via Kolmogorov–Smirnov tests. Categorical variables were summarized as frequency (percentage). The between-group differences among variables were analyzed by one-way analysis of variance (ANOVA) or the Kruskal–Wallis test, and Pearson's chi-squared test or Fisher's exact test for continuous and categorical variables, as appropriate. Univariable logistic regression identified potential confounding factors affecting CPR. The multivariable logistic regression model was then adopted to assess the association between the timing of oocyte retrieval and CPR, with results reported as adjusted odds ratios (aORs) with 95% confidence intervals (CIs). A two-tailed *P*-value < 0.05 was considered statistically significant. Data visualization was performed with R software (version 4.3.3) utilizing the ggplot2 and forestplot package for graphical representation.

## Results

### Overview of the study population

Figure 1 illustrates the patient screening flowchart. Since blastocyst transfers constituted only a minor proportion (68 cycles, 0.82%) of fresh cycles in our reproductive center, we exclusively analyzed fresh cleavage-stage embryo transfer cycles. A total of 8,200 eligible patients were included in the final analysis. The mean age of the participants was 30.45 ± 4.24 years, and the average BMI was 22.15 ± 2.34 kg/m^2^.

### Baseline characteristics of fresh cycles

**Fig. 2:**
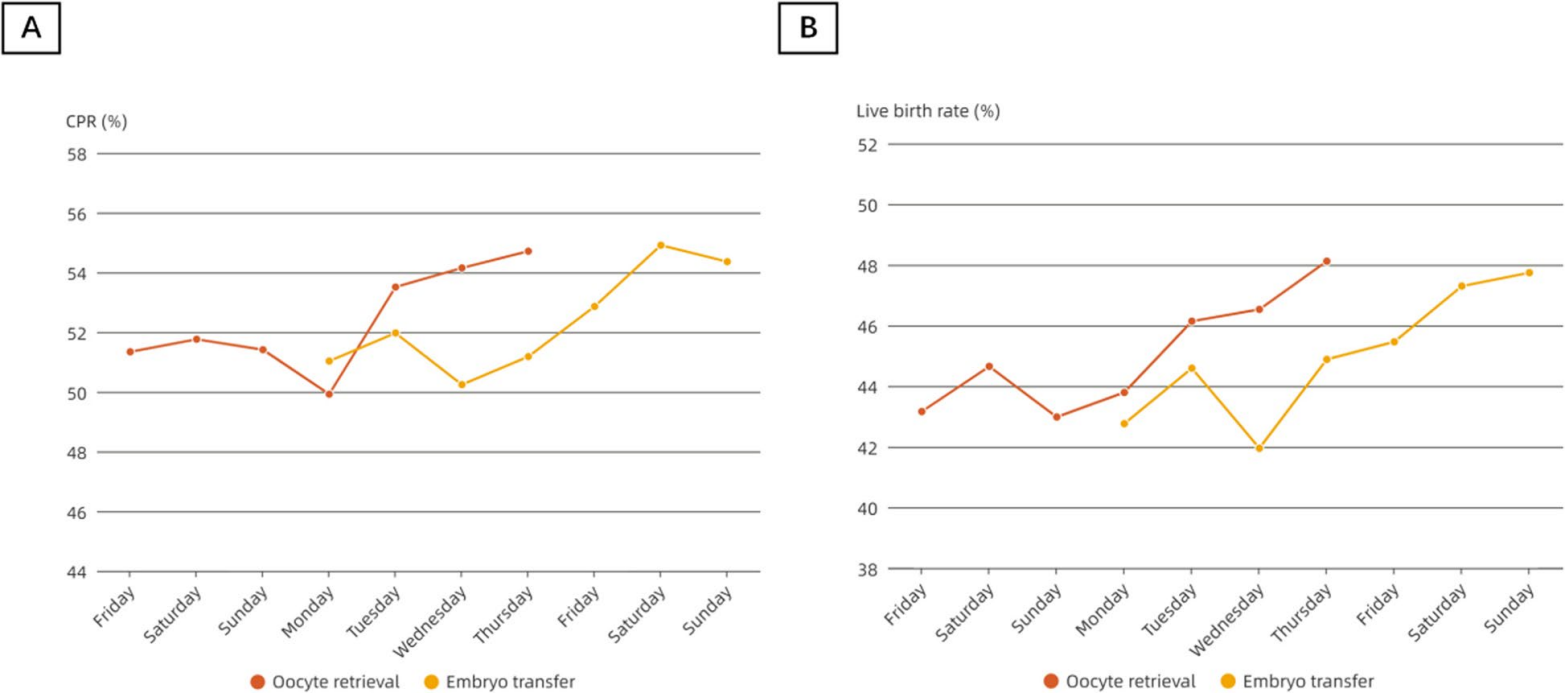
Trajectory of weekly variations in CPR and live birth rate, grouped by day of oocyte retrieval and embryo transfer. **A** The trajectory of weekly variations in CPR grouped by day of oocyte retrieval and embryo transfer; **B** The trajectory of weekly variations in live birth rate grouped by day of oocyte retrieval and embryo transfer. Abbreviation: CPR clinical pregnancy rate

In fresh ART cycles, oocyte retrieval and embryo transfer represent the two most critical procedures. Initially, we delineated the weekly variations in CPR and live birth rate stratified by the day of the week for both oocyte retrievals and embryo transfer. Notably, the fluctuation patterns observed in embryo transfer subgroups mirrored those of oocyte retrieval (Fig. 2; Table S1), indicating that the timing of embryo transfer is intrinsically determined by retrieval scheduling in fresh cycles. Consequently, we classified the fresh cycle cohorts primarily based on the day of oocyte retrieval in the subsequent analysis.

The 8,200 fresh cycles with cleavage-stage embryos transferred were categorized into three groups according to the day of oocyte retrieval: Weekend Group (Saturday and Sunday, *n* = 2,219); Near-Weekend Group (Friday and Monday, *n* = 2,435); and Midweek Group (Tuesday, Wednesday, and Thursday, *n* = 3,546). Baseline characteristics of the three groups were depicted in Table 1. No significant differences were found in most demographic and clinical characteristics such as age, BMI, duration of infertility, gravidity, parity, education level, etc. However, specific parameters revealed statistically significant disparities among the groups.

**Table 1:** Baseline characteristics of participants who underwent fresh ET cycles

Characteristics	Group 1: Weekend Group (*N* = 2219)	Group 2: Near-Weekend Group (*N* = 2435)	Group 3: Midweek Group (*N* = 3546)	*P* value
Age, years	30.48 ± 4.29	30.55 ± 4.20	30.36 ± 4.23	0.246
BMI, kg/m^2^	22.14 ± 2.36	22.10 ± 2.29	22.18 ± 2.37	0.474
Duration of infertility, years	3 (2, 5)	3 (2, 5)	3 (2, 5)	0.131
Gravidity, *n* (%)				0.479
0	1057 (47.63%)	1150 (47.23%)	1622 (45.74%)	
1	544 (24.52%)	626 (25.71%)	935 (26.37%)	
> = 2	618 (27.85%)	659 (27.06%)	989 (27.89%)	
Parity, *n* (%)				0.845
0	1757 (79.18%)	1919 (78.81%)	2785 (78.54%)	
> = 1	462 (20.82%)	516 (21.19%)	761 (21.46%)	
Education level, *n* (%)				0.668
Primary school and below	120 (5.41%)	138 (5.67%)	204 (5.75%)	
Secondary and High Schools	1336 (60.21%)	1448 (59.47%)	2167 (61.11%)	
College and above	763 (34.38%)	849 (34.86%)	1175 (33.14%)	
Occupational status, *n* (%)				0.762
Employed	802 (36.14%)	893 (36.67%)	1274 (35.93%)	
Self-employed	291 (13.11%)	346 (14.21%)	509 (14.35%)	
Freelance	340 (15.32%)	357 (14.66%)	506 (14.27%)	
Housewife	786 (35.43%)	839 (34.46%)	1257 (35.45%)	
Occupational exposure^*^, *n* (%)				0.883
Unexposed occupation	2055 (92.61%)	2264 (92.98%)	3293 (92.87%)	
Exposed occupation	164 (7.39%)	171 (7.02%)	253 (7.13%)	
Family residence^#^, *n* (%)				0.741
Changsha City	197 (8.88%)	215 (8.83%)	298 (8.40%)	
Outside Changsha and within Hunan Province	1334 (60.12%)	1434 (58.89%)	2092 (59.00%)	
Outside Hunan Province	688 (31.00%)	786 (32.28%)	1156 (32.60%)	
Main indication for ART, *n* (%)				0.011
Tubal factor	1510 (68.05%)	1669 (68.54%)	2428 (68.47%)	
Ovulation disorders	263 (11.85%)^b^	255 (10.47%)	321 (9.05%)^b^	
DOR	57 (2.57%)	63 (2.59%)	91 (2.57%)	
Male factor	279 (12.57%)	338 (13.88%)	491 (13.85%)	
Others^†^	110 (4.96%)	110 (4.52%)^c^	215 (6.06%)^c^	
Basal FSH, mIU/ml	6.50 (5.46, 7.74)	6.40 (5.41, 7.56)	6.44 (5.45, 7.76)	0.688
Basal LH, mIU/ml	4.94 (3.68, 6.73)	4.95 (3.60, 6.71)	5.03 (3.70, 6.70)	0.521
Basal E2, pg/ml	33.47 (25.30, 45.10)	34.20 (25.50, 43.40)	34.04 (26.06, 44.92)	0.531
Basal T, ng/ml	0.25 (0.17, 0.35)	0.25 (0.16, 0.34)	0.24 (0.16, 0.33)	0.098
AMH, ng/ml	3.35 (1.95, 5.58)	3.38 (2.03, 5.53)	3.34 (1.99, 5.48)	0.762
TSH, µIU/ml	2.17 (1.55, 3.10)	2.15 (1.51, 3.03)	2.16 (1.51, 3.10)	0.300
TG, mmol/l	1.10 (0.79, 1.56)	1.07 (0.81, 1.48)	1.07 (0.79, 1.48)	0.544
TC, mmol/l	4.64 (4.12, 5.21)	4.65 (4.12, 5.17)	4.60 (4.10, 5.22)	0.158
HDL-C, mmol/l	1.39 (1.18, 1.60)	1.38 (1.19, 1.61)	1.37 (1.18, 1.60)	0.819
LDL-C, mmol/l	2.78 (2.32, 3.23)	2.75 (2.32, 3.20)	2.71 (2.30, 3.20)	0.089
FBS, mmol/l	5.28 (5.01, 5.57)	5.28 (5.01, 5.57)	5.28 (5.02, 5.56)	0.287
FINS, µU/ml	9.79 (6.96, 13.35)	9.47 (6.93, 12.94)	9.45 (6.77, 13.16)	0.479
AFC, *n* (%)				0.231
1–6	249 (11.22%)	261 (10.27%)	384 (10.83%)	
7–12	715 (32.22%)	866 (35.56%)	1242 (35.03%)	
13–24	789 (35.56%)	806 (33.10%)	1222 (34.46%)	
> 24	466 (21.00%)	502 (20.62%)	698 (19.68%)	
Insemination, *n* (%)				0.184
IVF	1657 (74.67%)	1801 (73.96%)	2634 (74.28%)	
ICSI	385 (17.35%)	437 (17.95%)	671 (18.92%)	
IVF + ICSI	177 (7.98%)	197 (8.09%)	241 (6.80%)	
Endometrial thickness on hCG Day, mm	10.81 ± 2.14^b^	10.88 ± 2.16	10.98 ± 2.24^b^	0.021
Endometrial type, *n* (%)				0.443
A	958 (43.17%)	1140 (46.82%)	1552 (43.77%)	
B	1121 (50.52%)	1162 (47.72%)	1779 (50.17%)	
C	140 (6.31%)	133 (5.46%)	215 (6.06%)	
No. of embryos transferred, *n*	1.83 ± 0.38	1.85 ± 0.36	1.85 ± 0.36	0.113
Quality of embryos transferred, *n* (%)				0.005
Good-quality	3518/4060 (86.65%)^b^	3976/4507 (88.22%)	5809/6546 (88.74%)^b^	
Suboptimal quality	542/4060 (13.35%)^b^	531/4507 (11.78%)	737/6546 (11.26%)^b^	
Physician title of oocyte retrieval, *n* (%)				0.870
Associate chief physician and above	1033 (46.55%)	1117 (45.87%)	1648 (46.47%)	
Attending physician	1186 (53.45%)	1318 (54.13%)	1898 (53.53%)	
Physician gender of oocyte retrieval, *n* (%)				0.041
Female	1395 (62.87%)^a^	1443 (59.26%)^a^	2168 (61.14%)	
Male	824 (37.13%)^a^	992 (40.74%)^a^	1378 (38.86%)	
Physician title of embryo transfer, *n* (%)				< 0.001
Associate chief physician and above	1835 (82.69%)^a,b^	1884 (77.37%)^a^	2722 (76.76%)^b^	
Attending physician	384 (17.31%)^a,b^	551 (22.63%)^a^	824 (23.24%)^b^	
Physician gender of embryo transfer, *n* (%)				0.039
Female	1589 (71.67%)^a^	1822 (74.83%)^a^	2619 (73.86%)	
Male	630 (28.39%)^a^	613 (25.17%)^a^	927 (26.14%)	

Data presented as mean ± SD or median and interquartile rangeor for continuous variables, and *n* (%) for categorical variables

*Abbreviation*: *BMI* body mass index, *ART* assisted reproductive technology, *DOR* diminished ovarian reserve, *FSH* follicle stimulating hormone, *LH* luteinizing hormone, *E2* estradiol, *T* testosterone, *AMH* anti-Müllerian hormone, *TSH* thyroid-stimulating hormone, *TG* triglycerides, *TC* total cholesterol, *HDL-C* high-density lipoprotein cholesterol, *LDL-C* low-density lipoprotein cholesterol, *FBS* fasting blood sugar, *FINS* fasting insulin, *AFC* antral follicle count, *IVF* in vitro fertilization, *ICSI* intracytoplasmic sperm injection, *hCG* human chorionic gonadotropin

^*^Exposure of various hazardous agents within work environment, which might pose health risks (e.g., manicurist, doctors, nurses, electronic assembly workers, etc

^#^The Xiangya Hospital of CSU is located in Changsha, Hunan Province, a province in the south-central part of China

^†^Other ART indications refer to patients seeking treatment due to factors such as endometriosis, unexplained infertility, and recurrent miscarriages, etc

^a^Group 1 vs. Group 2: *P* < 0.05

^b^Group 1 vs. Group 3: *P* < 0.05

^c^Group 2 vs. Group 3: *P* < 0.05

**Fig. 3:**
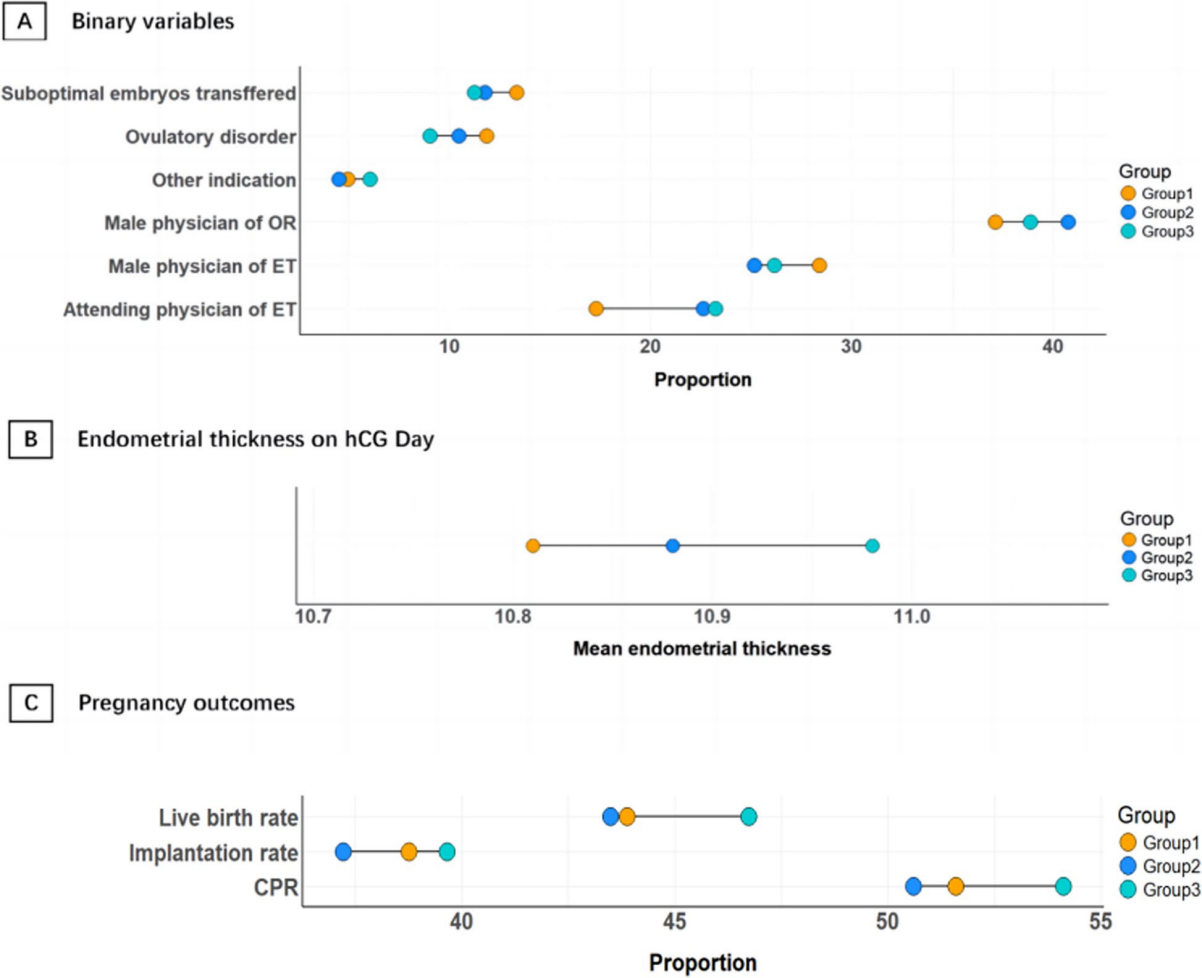
Significant differences in baseline characteristics and pregnancy outcomes among the three groups who underwent fresh ET cycles. Abbreviation: OR oocyte retrieval, ET embryo transfer, hCG human chorionic gonadotropin, CPR clinical pregnancy rate. **A** Binary variables in baseline characteristics. **B** Endometrial thickness. **C** Pregnancy outcomes

An elevated proportion of ovulation disorders (11.85%, vs. 9.05%, *p* = 0.001) and lower proportion of good-quality embryos (86.65% vs. 88.74%, *p* = 0.001) were observed in Weekend Group relative to Midweek Group. Meanwhile, the Weekend Group exhibited a reduced endometrial thickness on hCG day compared to the Midweek Group (10.81 ± 2.14 mm vs. 10.98 ± 2.24 mm, *p* = 0.018). The Weekend Group had a lower proportion of male physicians on the day of oocyte retrieval compared to Near-Weekend Group (37.13% vs. 40.74%, *p* = 0.013), but a higher proportion of male physicians on the day of embryo transfer compared to the Near-Weekend Group (28.39% vs. 25.17%, *p* = 0.014) (Fig. 3). And the Weekend Group had the lowest ratio of attending physician on the day of oocyte retrieval among the three groups (17.31% vs. 22.63% vs. 23.24%, *p* < 0.001) (Table 1).

### Pregnancy outcomes among the three groups who underwent fresh ET cycles

When contrasted with the midweek group, the near-weekend group demonstrated a significant inferior outcomes in terms of CPR (50.60% vs. 54.12%, *p* = 0.007), implantation rate (37.23% vs. 39.66%, *p* = 0.010) and live birth rate (43.49% vs. 46.73%, *p* = 0.014) (Table 2, Fig. 3). Similarly, the weekend group exhibited lower rates of clinical pregnancy, implantation and live birth compared to the midweek group, while the differences were not statistically significant (CPR: 51.60% vs. 54.12%; Implantation rate: 38.77% vs. 39.66%; Live birth rate: 43.88% vs. 46.73%, all *p* > 0.05). No statistically significant differences were observed among the three groups in other secondary pregnancy outcomes, including β-hCG positivity rate, biochemical pregnancy rate, ectopic pregnancy rate, miscarriage rate, or ongoing pregnancy rate (all *p* > 0.05).

**Table 2:** Pregnancy outcomes of the three groups who underwent fresh ET cycles

Pregnancy outcomes	Group 1: Weekend Group (*N* = 2219)	Group 2: Near-Weekend Group (*N* = 2435)	Group 3: Midweek Group *(N* = 3546)	*P* value
β-hCG positivity rate, *n* (%)	1382 (62.28%)	1496 (61.44%)	2250 (63.45%)	0.274
Biochemical pregnancy rate, *n* (%)	192 (8.65%)	220 (9.03%)	283 (7.98%)	0.334
CPR, *n* (%)	1145 (51.60%)	1232 (50.60%)^c^	1919 (54.12%)^c^	0.019
Implantation rate, *n* (%)	1574/4060 (38.77%)	1678/4507 (37.23%)^c^	2596/6546 (39.66%)^c^	0.036
Ectopic pregnancy rate, *n* (%)	50 (2.25%)	48 (1.97%)	52 (1.47%)	0.079
Miscarriage rate, *n* (%)	156/1123 (13.89%)	162/1209 (13.40%)	237/1885 (12.57%)	0.808
Ongoing pregnancy rate, *n* (%)	1021/2197 (46.06%)	1114/2412 (46.19%)	1720/3512 (48.97%)	0.059
Live birth rate, *n* (%)	964/2197 (43.88%)	1049/2412 (43.49%)^c^	1641/3512 (46.73%)^c^	0.023
Multiple pregnancy rate, *n* (%)	300/2197 (13.65%)	333/2412 (13.81%)	493/3512 (14.04%)	0.916
Loss follow-up, *n* (%)	22 (0.99%)	23 (0.94%)	34 (0.96%)	0.986

Data presented as *n* (%) for categorical variables

*Abbreviation*: *CPR* clinical pregnancy rate

^c^Group 2 vs. Group 3: *P* < 0.05

### Multivariable regression identifies retrieval timing as an independent CPR predictor

To identify the confounding factors that might influence the association between the timing of oocyte retrieval and CPR, we initially conducted univariate logistic regression analyses. This preliminary step revealed variables such as age, duration of infertility, gravidity, parity, current career status, main indication for ART, basal follicle stimulating hormone (FSH), E2, testosterone (T), anti-Müllerian hormone (AMH), antral follicle count (AFC), endometrial thickness on hCG day, endometrial type, number of embryos transferred and good-quality embryos transferred as potential confounders (Table 3). Subsequently, these variables were integrated into a multivariate logistic regression model, thereby enabling a more precise evaluation of the relationship between the timing of oocyte retrieval and CPR.

**Table 3:** Univariate and multivariate logistic regression analysis of variables influencing CPR in fresh cycles

Variable	Univariate Binary Logistic Regression		Multivariate Logistic Regression	
	Crude OR (95% CI)	*P* value	Adjusted OR (95% CI)	*P* value
Group		0.019		0.035
Midweek Group	Reference		Reference	
Weekend Group	0.904 (0.813, 1.005)	0.062	0.900 (0.781, 1.037)	0.144
Near-Weekend Group	0.868 (0.783, 0.963)	0.007	0.836 (0.728, 0.960)	0.011
Age, years	0.949 (0.939, 0.959)	0.000	0.958 (0.942, 0.975)	0.000
BMI, kg/m^2^	1.005 (0.987, 1.024)	0.578		
Duration of infertility, years	0.984 (0.970, 0.998)	0.028	1.015 (0.994, 1.036)	0.162
Gravidity, *n*	0.916 (0.886, 0.948)	0.000	1.012 (0.957, 1.072)	0.670
Parity, *n*	0.785 (0.717, 0.861)	0.000	0.959 (0.829, 1.110)	0.576
Education level		0.090		
Primary school and below	Reference			
Secondary and High Schools	1.128 (0.932, 1.365)	0.216		
College and above	1.215 (0.998, 1.480)	0.052		
Current career status		0.004		0.108
Employed	Reference		Reference	
Self-employed	0.824 (0.719, 0.944)	0.005	0.832 (0.688, 1.007)	0.058
Freelance	0.915 (0.800, 1.046)	0.192	0.860 (0.724, 1.023)	0.089
Housewife	1.044 (0.942, 1.157)	0.408	0.988 (0.860, 1.137)	0.871
Occupational exposure				
Unexposed occupation	Reference			
Exposed occupation	0.978 (0.827, 1.157)	0.793		
Family residence		0.404		
Changsha City	Reference			
Outside Changsha and within Hunan Province	0.929 (0.793, 1.088)	0.360		
Outside Hunan Province	0.895 (0.758, 1.057)	0.190		
Main indication for ART		0.000		0.134
Tubal factor	Reference		Reference	
Ovulation disorders	1.186 (1.025, 1.372)	0.022	1.098 (0.871, 1.386)	0.428
DOR	0.610 (0.460, 0.808)	0.001	1.015 (0.710, 1.453)	0.933
Male factor	1.131 (0.994, 1.287)	0.063	1.052 (0.879, 1.260)	0.581
Others	1.270 (1.043, 1.547)	0.017	1.418 (1.086, 1.852)	0.010
Basal FSH, mIU/ml	0.957 (0.937, 0.977)	0.000	1.014 (0.986, 1.043)	0.338
Basal LH, mIU/ml	1.013 (1.000, 1.025)	0.050		
Basal E2, pg/ml	0.997 (0.995, 0.999)	0.010	0.997 (0.994, 1.000)	0.056
Basal T, ng/ml	1.694 (1.251, 2.295)	0.001	1.618 (1.064, 2.461)	0.024
AMH, ng/ml	1.038 (1.022, 1.055)	0.000	0.996 (0.976, 1.017)	0.703
TSH, µIU/ml	1.008 (0.984, 1.033)	0.532		
TG, mmol/l	0.969 (0.912, 1.028)	0.296		
TC, mmol/l	0.975 (0.918, 1.036)	0.413		
HDL-C, mmol/l	1.122 (0.965, 1.305)	0.133		
LDL-C, mmol/l	0.965 (0.897, 1.039)	0.348		
FBS, mmol/l	0.951 (0.855, 1.057)	0.352		
FINS, µU/ml	1.004 (0.995, 1.013)	0.418		
AFC		0.000		0.003
1–6	Reference		Reference	
7–12	1.590 (1.364, 1.854)	0.000	1.311 (1.057, 1.625)	0.014
13–24	1.931 (1.656, 2.251)	0.000	1.522 (1.215, 1.908)	0.000
> 24	2.053 (1.739, 2.422)	0.000	1.465 (1.119, 1.920)	0.006
Insemination		0.050		
IVF	Reference			
ICSI	0.994 (0.887, 1.113)	0.911		
IVF + ICSI	0.813 (0.689, 0.960)	0.015		
Endometrial thickness on hCG Day, mm	1.108 (1.086, 1.131)	0.000	1.117 (1.087, 1.147)	0.000
Endometrial type		0.002		0.007
A	Reference			
B	0.927 (0.848, 1.014)	0.097	0.877 (0.778, 0.989)	0.032
C	0.722 (0.598, 0.873)	0.001	0.695 (0.537, 0.900)	0.006
No. of embryos transferred, *n*	2.170 (1.920, 2.453)	0.000	1.254 (1.039, 1.514)	0.019
No. of good-quality embryos transferred, *n*	1.934 (1.800, 2.079)	0.000	1.890 (1.690, 2.112)	0.000
Physician title of oocyte retrieval				
Associate chief physician and above	Reference			
Attending physician	1.062 (0.973, 1.158)	0.176		
Physician gender of oocyte retrieval				
Female	Reference			
Male	0.934 (0.854, 1.021)	0.131		
Physician title of embryo transfer				
Associate chief physician and above	Reference			
Attending physician	0.961 (0.865, 1.068)	0.466		
Physician gender of embryo transfer				
Female	Reference			
Male	0.939 (0.852, 1.036)	0.213		

*Abbreviation*: *BMI* body mass index, *ART* assisted reproductive technology, *DOR* diminished ovarian reserve, *FSH* follicle stimulating hormone, *LH* luteinizing hormone, *E2* estradiol, *T* testosterone, *AMH* anti-Müllerian hormone, *TSH* thyroid-stimulating hormone, *TG* triglycerides, *TC* total cholesterol, *HDL-C* high-density lipoprotein cholesterol, *LDL-C* low-density lipoprotein cholesterol, *FBS* fasting blood sugar, *FINS* fasting insulin, *AFC* antral follicle count, *IVF* in vitro fertilization, *ICSI* intracytoplasmic sperm injection, *hCG* human chorionic gonadotropin, *OR* odds ratio, *95%CI* 95% confidence interval

**Fig. 4:**
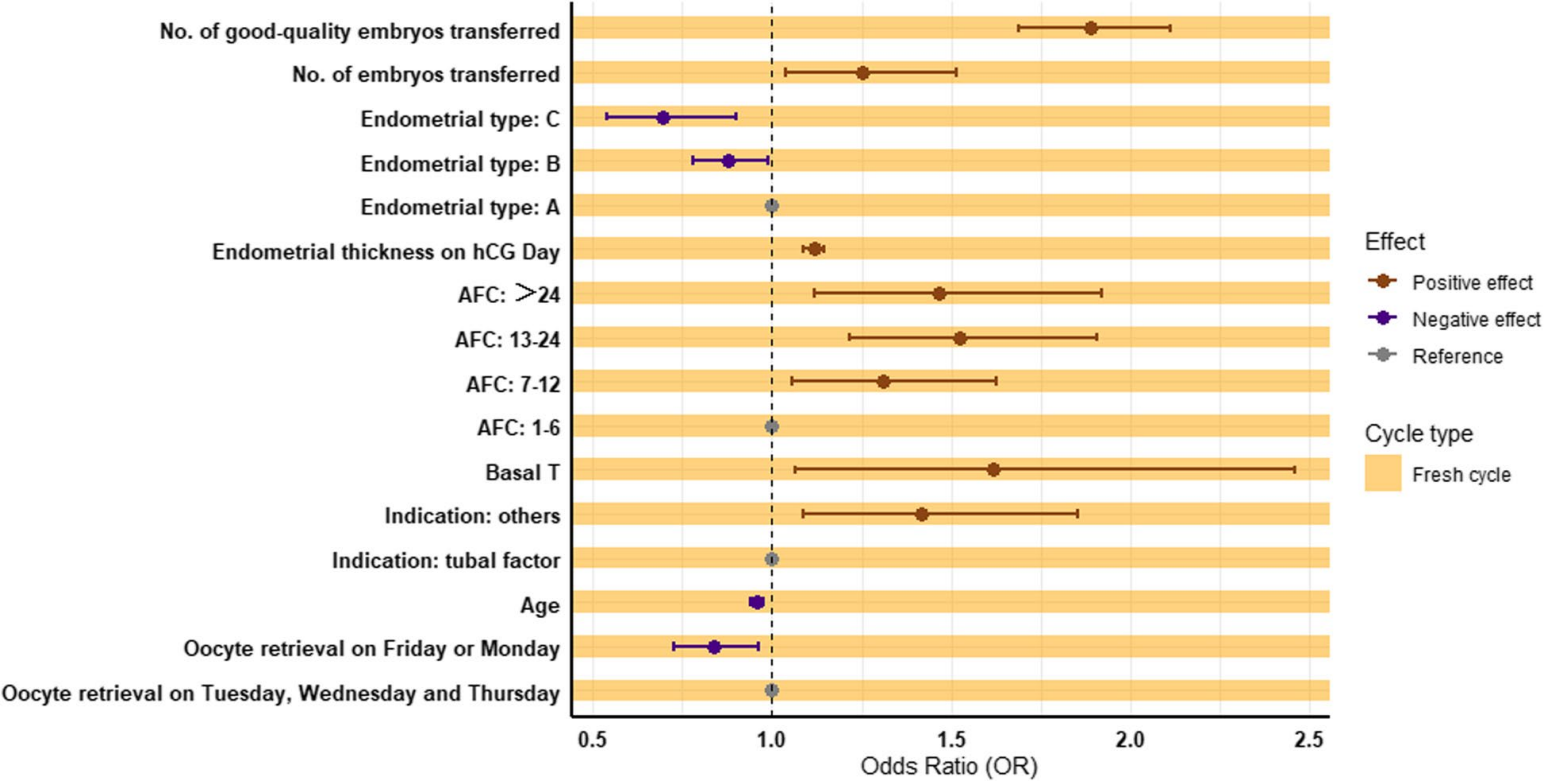
Multivariate logistic regression analysis of variables influencing CPR in the fresh cycles. Abbreviation: AFC antral follicle count, hCG human chorionic gonadotropin, T testosterone

Compared to midweek retrievals, oocyte retrieval performed near weekends had significantly lower odds of achieving a CPR after adjusting for confounders (adjusted OR 0.836, 95%CI 0.728–0.960, *p* = 0.011, Table 3, Fig. 4). The adjusted odds ratios indicated that near-weekend oocyte retrievals were associated with an approximately 16% reduction in the likelihood of achieving clinical pregnancy compared to midweek retrievals.The Weekend Group also displayed lower odds of CPR compared to the Midweek Group, although this difference was not statistically significant (adjusted OR: 0.900, 95%CI 0.781–1.037, *p* = 0.144).

## Discussion

The present study aimed to evaluate the presence of a weekend effect in the first fresh embryo transfer cycle at a single high-volume center that provides year-round, seven-day services. In this study, compared to midweek oocyte retrievals, those scheduled near the weekend (Friday or Monday) were associate with worse pregnancy outcomes. Although the CPR for weekend oocyte retrieval was also lower than that of midweek, the difference was not statistically significant.

Since the mid-to-late twentieth century, many countries in Europe and North America have progressively enacted legislation to institutionalize the two-day weekend system, which standardizes a 40-h workweek with rest days on Saturday and Sunday. In China, this system was formally implemented nationwide in 1995. The primary objective of the two-day weekend is to ensure adequate and reasonable rest for workers, reflecting a human-centric philosophy. By allowing employees to recover physically and mentally, it enhances their overall well-being and productivity, thereby fostering harmonious labor relations.

However, despite decades of widespread adoption, certain professions—due to their inherent operational demands—remain exempt from this system. A notable example is medical professionals, whose critical and round-the-clock responsibilities often prevent them from benefiting from the standard two-day weekend. Consequently, this has given rise to research on cyclical variations in work efficiency. In 2001, Bell et al. first reported in *The New England Journal of Medicine* that patients admitted on weekends exhibited higher mortality rates than those admitted on weekdays—a landmark study that sparked widespread discourse on the “weekend effect” [17].

Given the impact of the weekend effect, clinicians have further observed its influence on patient outcomes in the days adjacent to weekends. For instance, a recent multi-center retrospective study demonstrated that patients undergoing surgery immediately before weekends (typically Fridays) faced significantly higher risks of complications, readmissions, and mortality compared to those treated after weekends (typically Mondays) [18]. This suggests that the persistence of the weekend effect extends to near-weekends, further underscoring the critical need to understand this phenomenon and its implications for patient care.

During ovarian stimulation cycles for fresh embryo transfer in ART, length of ovarian stimulation is often unpredictable. Therefore, we do not intentionally avoid weekend oocyte retrievals, meaning we neither advance them to Friday nor delay them to Monday. Regarding oocyte retrieval on weekdays versus weekends, in this study, fresh embryo transfer outcomes following weekend oocyte retrievals were comparable to those after weekday retrievals (CPR: 51.60% vs 52.68%, *p* = 0.383; live birth rate: 43.88% vs 45.41%, *p* = 0.218). These findings align with previous ART studies demonstrating no significant weekend-weekday disparity in reproductive outcomes [10, 12]. These suggest that ART outcomes appear unaffected by the so-called “weekend effect”.

However, our study identified a novel “near-weekend effect”. Compared to midweek oocyte retrievals, retrievals performed near weekends were associated with significantly lower rates of clinical pregnancy, implantation and live birth. Most demographic and clinical characteristics were comparable among the three groups, except for the main ART indication, endometrial thickness on the hCG day, and quality of embryos transferred, etc. After adjusting for confounders in the multivariate logistic regression analysis, the Near-Weekend Group demonstrated lower odds of achieving a CPR (adjusted OR 0.836, 95%CI 0.728–0.960, *p* = 0.011).

Contrary to our results, Ben-Chetrit et al. failed to find significant differences in outcomes among patients who underwent oocyte retrieval on Monday or Friday versus midweek [19]. Their study involved only a “flare-up” protocol using a GnRH agonist and had a small sample size of just over 500. The GnRH antagonist protocol offers greater patient friendly in IVF experience compared to the GnRH agonist regimen [20]. Tremellen et al. compared outcomes between ideal and non-ideal (intentionally advance or delay) oocyte retrievals conducted on Monday or Friday [21]. Despite differences in the duration of Gn stimulation, the numbers of oocytes retrieved, and the number of embryos transferred between the ideal and non-ideal group, these did not translate into any differences in pregnancy outcomes. Combined with the result of Setti et al. who directly focused on whether performing oocyte retrieval on a weekday vs. weekend impacts pregnancy outcomes [10], the available evidence suggests that the specific day of oocyte retrieval during the week may not significantly influence ART success.

It has been claimed that patients admitted on weekends tend to be in poorer health and are often considered more urgent than those treated on weekdays [7]. Furthermore, potential compromise in care quality and limited resource availability during weekends may result in suboptimal results [6]. However, neither of these situations appears to apply to our current setting. We assumed that the subtle psychological variations in both doctors and patients as they approach the weekend, or immediately after the weekend should be taken into consideration. For example, non-restful weekends (despite having a rotational morning shift system) may lead to diminished performance of doctors performing oocyte retrievals on Mondays, particularly given that our center provides uninterrupted seven-day services.

To the best of our knowledge, this is the first large-scale study on the weekend effect in the field of ART conducted in a Chinese population. For the first time, we demonstrated the presence of a “near-weekend effect”, rather than a classic “weekend effect”, in the first fresh embryo transfer cycles. A sample size of 8,200 cycles provides substantial statistical power. The comprehensive data collection ensured detailed and systematic documentation of clinical practices, procedural timings, and pregnancy outcomes, thereby bolstering the reliability of the findings. Subsequently, we employed both univariate and multivariate logistic regression analyses with adjustment for potential confounders, which strengthens the validity of the observed association between procedural timing and CPR.

We hypothesize that this may be related to accumulated physician fatigue over the course of the workweek, variations in cognitive workload that affect clinical decision-making, and potential subconscious chronobiological biases in procedure execution. Our findings suggest that hospitals should optimize resource allocation to ensure patients receive equally attentive care from well-rested physicians on any day of the week. This study highlights the necessity of implementing structured staff rotation systems to maintain consistent clinical quality.

However, the study also has several limitations that should be considered when interpreting the findings. Firstly, the retrospective cohort design is prone to biases inherent in retrospective data collection and analysis, such as selection bias and information bias. Secondly, as a single-center study, the findings might not be entirely generalizable to other settings with different operational practices or patient demographics. Thirdly, the extended study period (2014–2022) encompassed several advancements in ART (e.g., the standardization of embryo culture conditions, improvements in vitrification techniques, and evolving ovarian stimulation protocols), which may have introduced temporal confounders. Lastly, the absence of randomization means that unmeasured confounders might still exist, potentially influencing the observed associations between the timing of procedures and pregnancy outcomes. Moving forward, multi-center collaborative prospective studies will be essential to validate and extend these findings in larger clinical cohorts, thereby mitigating potential biases arising from variations in clinical practice or patient demographics.

## Conclusions

The timing of oocyte retrieval near weekends negatively impacts the CPR in fresh cycles. Understanding these dynamics is crucial, as it underscores the need for consistent clinical vigilance—regardless of the day of the week—to optimize both pregnancy success rates and patient satisfaction.

## Supplementary Information

Supplementary Material 1.

## Data Availability

The datasets used and/or analyzed during the current study are available from the corresponding author on reasonable request.

## Abbreviations

AFC: Antral follicle count
AMH: Anti-Müllerian hormone
ANOVA: Analysis of variance
ART: Assisted reproductive technology
BMI: Body mass index
CE: Chronic endometritis
CI: Confidence interval
CPR: Clinical pregnancy rate
CSU: Central South University
DM: Diabetes mellitus
DOR: Diminished ovarian reserve
E2: Estradiol
ET: Embryo transfer
EMs: Endometriosis
FBS: Fasting blood sugar
FINS: Fasting insulin
FSH: Follicle stimulating hormone
Gn: Gonadotrophin
GnRH: Gonadotrophin-releasing hormone
hCG: Human chorionic gonadotropin
HDL-C: High-density lipoprotein cholesterol
HRT: Hormone replacement therapy
ICSI: Intracytoplasmic sperm injection
IUA: Intrauterine adhesion
IVF: In vitro fertilization
LDL-C: Low-density lipoprotein cholesterol
LH: Luteinizing hormone
MII: Metaphase II
OHSS: Ovarian hyperstimulation syndrome
OR: Odds ratio
P4: Progesterone
PPOS: Progestin-primed ovarian stimulation
SD: Standard deviation
T: Testosterone
TC: Total cholesterol
TG: Triglycerides
TSH: Thyroid-stimulating hormone
