# Supplementary material for: Does the weekend matter? evaluating the weekend effect on pregnancy outcomes in first-cycle fresh IVF/ICSI embryo transfers: a retrospective study
Source: BMC Pregnancy Childbirth. 2025 Jul 3;25:708. doi: 10.1186/s12884-025-07783-x (PMC12224509; doi:10.1186/s12884-025-07783-x)
Supplement: Supplementary file 1 — Supplementary Material 1. [file 12884_2025_7783_MOESM1_ESM.docx]

| **Table S1.** Outcome of oocyte retrieval and embryo transfer by day of the week. | | | | | | | |
| --- | --- | --- | --- | --- | --- | --- | --- |
| Outcomes | Grouped according to the day of the week on which the oocyte retrieval occurred | | | | | | |
|  | Monday | Tuesday | Wednesday | Thursday | Friday | Saturday | Sunday |
|  | N=1274 | N=1155 | N=1265 | N=1126 | N=1161 | N=1194 | N=1025 |
| CPR | 49.92% | 53.51% | 54.15% | 54.71% | 51.34% | 51.76% | 51.41% |
|  | (636/1274) | (618/1155) | (685/1265) | (616/1126) | (596/1161) | (618/1194) | (527/1025) |
| Live birth rate | 43.79% | 46.14% | 46.53% | 48.12% | 43.16% | 44.65% | 42.98% |
|  | (554/1265) | (520/1127) | (584/1255) | (537/1116) | (495/1147) | (526/1178) | (438/1019) |
| Loss to follow-up^*^, n | 9 | 14 | 10 | 10 | 14 | 16 | 6 |
|  | Grouped according to the day of the week on which the embryo transfer occurred | | | | | | |
| Outcomes | Monday | Tuesday | Wednesday | Thursday | Friday | Saturday | Sunday |
|  | N=1209 | N=1191 | N=1029 | N=1270 | N=1135 | N=1253 | N=1113 |
| CPR | 51.03% | 51.97% | 50.24% | 51.18% | 52.86% | 54.91% | 54.36% |
|  | (617/1209) | (619/1191) | (517/1029) | (650/1270) | (600/1135) | (688/1253) | (605/1113) |
| Live birth rate | 42.76% | 44.59% | 41.95% | 44.88% | 45.46% | 47.30% | 47.74% |
|  | 511/1195 | 523/1173 | 430/1025 | 565/1259 | 511/1124 | 587/1241 | 527/1104 |
| Loss to follow-up^*^, n | 14 | 18 | 4 | 11 | 11 | 12 | 9 |
| Abbreviation: CPR clinical pregnancy rate.  ^*^All patients were followed up until 35 days post-embryo transfer for an ultrasound examination. Loss to follow-up occurred only in subsequent follow-ups. | | | | | | | |
